# An Unsupervised Learning-Based Spatial Co-Location Detection System from Low-Power Consumption Sensor †

**DOI:** 10.3390/s21144773

**Published:** 2021-07-13

**Authors:** David Ishak Kosasih, Byung-Gook Lee, Hyotaek Lim, Mohammed Atiquzzaman

**Affiliations:** 1 Department of Computer Engineering, Dongseo University, Busan 47011, Korea; ishakdavids@g.dongseo.ac.kr (D.I.K.); lbg@dongseo.ac.kr (B.-G.L.); 2 School of Computer Science, University of Oklahoma, Norman, OK 73019, USA; atiq@ou.edu

**Keywords:** spatial co-location detection, low-power consumption sensor, convolutional autoencoder, cloud computing, mobile application

## Abstract

Spatial co-location detection is the task of inferring the co-location of two or more objects in the geographic space. Mobile devices, especially a smartphone, are commonly employed to accomplish this task with the human object. Previous work focused on analyzing mobile GPS data to accomplish this task. While this approach may guarantee high accuracy from the perspective of the data, it is considered inefficient since knowing the object’s absolute geographic location is not required to accomplish this task. This work proposed the implementation of the unsupervised learning-based algorithm, namely convolutional autoencoder, to infer the co-location of people from a low-power consumption sensor data—magnetometer readings. The idea is that if the trained model can also reconstruct the other data with the structural similarity (SSIM) index being above 0.5, we can then conclude that the observed individuals were co-located. The evaluation of our system has indicated that the proposed approach could recognize the spatial co-location of people from magnetometer readings.

## 1. Introduction

A spatial co-location can be defined as a set of objects which co-exist in close geographic proximity [1]. Therefore, spatial co-location detection refers to the task of inferring the co-location of two or more objects in the geographic space [2,3]. This concept has been well developed in various fields, including, but not limited to, urban planning [4], transportation management for emission reduction [5], healthcare [6,7,8], crime analysis [9], and weather [10]. In particular, a spatial co-location detection system could be the key to control the spreading of infectious disease during an epidemic, or pandemic situation [11,12]. There are many sensors that can be deployed to accomplish this task, with global positioning systems (GPS) being the commonly used sensor [13,14].

Presently, mobile devices, such as smartphones and smartwatches, have emerged as one of the most common ways to perform location tracking-related tasks, including spatial co-location detection, with the human object. This is because the majority of people already carry these devices with them everywhere. More importantly, mobile devices, especially smartphones, are equipped with GPS and other sensors that could be used to accomplish location tracking-related tasks [15]. The common problems with this, however, are performance, privacy, and security [16,17,18]. In terms of performance, the impact of the sensors’ usage, particularly GPS, on the device’s battery life and accuracy are the two main problems, as also suggested by Nguyen et al. [6]. Although GPS may achieve the highest accuracy [19] as it directly collects one’s spatial location, it takes a toll on the device’s battery as it drains so much battery power [20] especially under weak signal strength [21].

In the concept of spatial co-location analysis or detection with the human object, however, one is not obliged to use GPS since this task does not require us to know the exact geographic position of the object, or, in other words, knowing if two or more individuals were in the same location within a specific time interval is more than sufficient. Research on finding the alternative to GPS for performing spatial co-location detection tasks was previously conducted. Analyzing magnetometer readings to detect co-location of people was proposed by Nguyen et al. [6]. A similar approach was also suggested in [22]. Combining the recent development of deep learning algorithms and magnetometer readings analysis to detect co-location of two or more individuals was suggested by Kosasih et al. [8]. Finally, the use of an accelerometer to replace GPS was proposed by Kuk et al. [23]. Although the new approaches [6,22,23] successfully performed co-location detection using low-power consumption sensors, they were restricted within limited areas. In addition, while the co-location detection technique proposed by Kosasih et al. [8] may alleviate previous limitations, it is rather difficult to find the appropriate threshold. These issues indicate that the previous approaches may still be ineffective for general use.

In this paper, we propose an unsupervised learning-based system that uses mobile magnetometer readings to infer the spatial co-location of people, similar to that of Kosasih et al. [8]. The main objectives of our approach are to use a low-power consumption sensor, reduce GPS data’s security issue, and make it adequate for general use. As has been investigated by Lin et al. [20] and Tawalbeh et al. [21], GPS drains so much battery power, especially under weak signal strength, such as in train and underground. By replacing GPS with the magnetometer, our system would consume less battery power as suggested by Nguyen et al. [6]. In fact, in the area where GPS suffers from weak signal strength, magnetometer readings actually achieve their highest accuracy, as also shown in [6]. Moreover, unlike GPS that collects plain geographic location (latitude, longitude, altitude), a magnetometer only measures magnetic field strength in a particular area, making it difficult for the adversary to infer the user’s actual position. Finally, our hypothesis is that if the threshold could also be uniformed, unlike those proposed by Kosasih et al. [8], the number of both false negative and false positive could be reduced, thus improving the accuracy.

In a nutshell, we proposed an unsupervised learning-based system implemented on the cloud to infer spatial co-location of the people from magnetometer readings. The concept of cloud computing would enable us to implement the algorithms that require expensive computation on the cloud, thus alleviating hardware limitations problem. The evaluation results of our system have shown that it manages to precisely differentiate co-located and non-co-located users. To summarize, this paper makes the following contributions:

1. Proposal of a cloud computing and unsupervised learning-based system for inferring spatial co-location of people from magnetometer data.
2. Performance evaluation and analysis of the convolutional-autoencoder model for spatial co-location detection.

The remainder of this paper is structured as follows. Section 2 outlines related work on spatial co-location detection. Section 3 provides the design rationale and architecture of our proposed system. Section 4 shows the performance evaluation through experimental results and analysis. Finally, in Section 5, we conclude our paper and discuss future work directions.

## 2. Related Work

One of the earliest work on using a magnetometer to infer co-location of people was proposed by [6]. Derivative dynamic time warping (DDTW) was implemented to match two magnetic trajectories. The evaluation of this system was focused on bus and train environments. Although this approach performed well in the overground and underground train, it was unreliable in the bus environment since the readings showed little spatial variation. This indicates that the proposed approach may also be difficult to be used outside the experiment environment set-up.

A magnetometer readings-based co-location detection was also proposed by Jeong et al. [22]; however, the Pearson correlation coefficient was implemented instead of DDTW to measure similarity between two readings. Similar to that of Nguyen et al. [6], the problem with this approach is also that n−1 times comparison process would be needed to perform spatial co-location detection task to *n* users’ data with one target, which can be ineffective and time-consuming.

Kosasih et al. [8] suggested the use of a vanilla autoencoder to infer spatial co-location of two or more individuals. The idea is that by training an autoencoder model on a magnetic trajectory, we can then just feed other magnetic trajectories to the trained model and analyze the mean squared error (MSE) values. A similar MSE value to the training MSE would indicate that magnetic readings from the other devices were also collected from the same location. Although this approach may improve the previous work, it can still be difficult to determine how close the test MSE to the train MSE such that it can be inferred that the users were co-located. In other words, it is rather impractical to define a proper threshold that would work in general.

An experiment on the carriage level co-location of people from accelerometer data was conducted by Kuk et al. [23]. Simple euclidean distance was used as a classifier for every two readings. There are two main problems with this approach. First, people’s movement will generate a lot of biases to the readings, thus lead to false negative results. Secondly, this approach cannot be employed in other environments, such as department stores or other common non-moving objects.

## 3. Proposed Method

As previously mentioned, we proposed an unsupervised learning-based system implemented on the cloud to detect spatial co-location of two or more individuals from low-power consumption sensor data, namely magnetometer readings. To perform this task, our system uses a convolutional autoencoder model and structural similarity (SSIM) index analysis to differentiate sensor readings.

### 3.1. System Model

**Figure 1:**
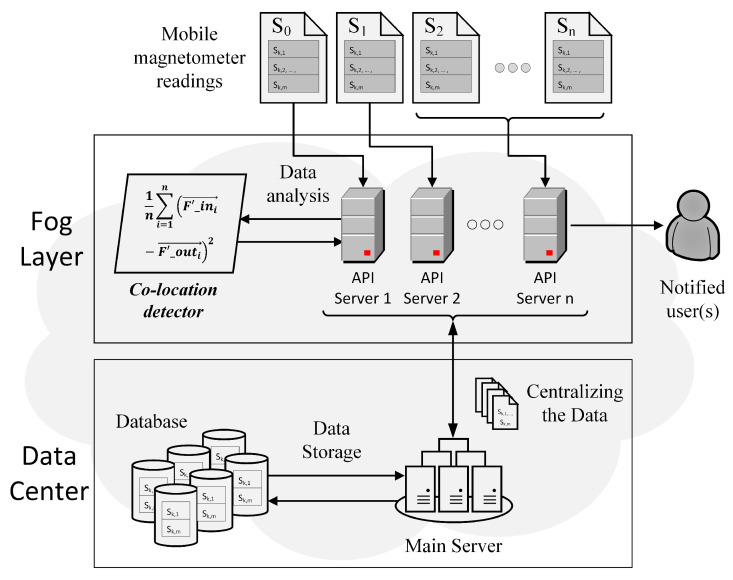
The concepts of fog computing and RESTful API server were implemented in our system. The primary purpose of using the fog layer is to improve the computational speed while still preserving low latency communications. The unsupervised learning technique for differentiating sensor readings was executed in the local server illustrated as the Co-location detector (parallelogram).

The general structure of our proposed system is illustrated in Figure 1. 3-axis magnetometer data are continuously collected by the users’ mobile phones in the background. Whenever an internet connection is available, the sensor readings will be uploaded to the nearby RESTful API server (see fog layer in Figure 1). The RESTful API server on this layer is responsible for maintaining server-client data communication, including handling the incoming data from the client and passing the data to the main server. After the sensor readings being passed to the main server (see data center layer in Figure 1), these data will then be saved inside the database for later use.

Suppose that we would like to know if one was spatially co-located with one or more other individuals, a notification can first be sent to the local or nearby RESTful API server by the subject’s mobile phone. Next, the local server notifies the main server by passing on the subject’s device information. The main server will then fetch the user’s magnetometer data as well as other users’ data from the database and send it back to the local server (fog layer). Upon receiving the magnetometer readings, the local server will perform an unsupervised co-location detection task inside an entity called a Co-location detector (see fog layer in Figure 1). An indicator will then be returned to the local server by the Co-location detector if one or more individuals are found to be spatially co-located with the subject. Finally, the local server will notify the corresponding individual(s)’ device for further use.

**Figure 2:**
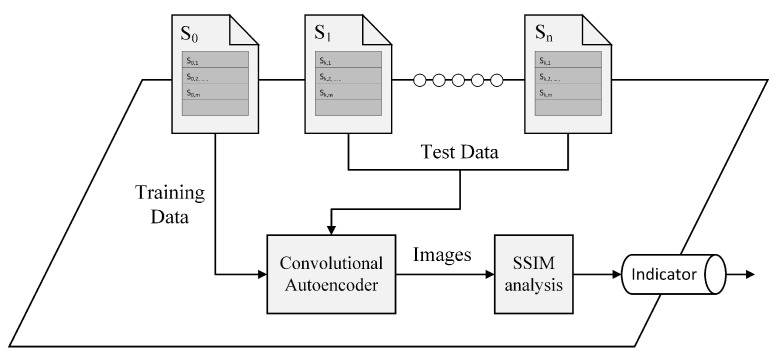
Spatial co-location analysis inside the Co-location detector. A convolutional autoencoder model is implemented in this main part to differentiate magnetometer readings.

The general procedures of our spatial co-location detection process inside the Co-location detector can be seen in Figure 2. Upon receiving magnetometer readings, it trains an unsupervised deep learning model, convolutional autoencoder, using the pre-processed subject’s mobile magnetometer data. After the model learns how to reconstruct the training data which belongs to the subject’s mobile phone, it will then try to reconstruct the test data, which eventually came from the other users’ mobile phones. SSIM [24,25] indexes analysis between the original and reconstructed test data can then be used to recognize the spatially co-located individuals (for a detailed procedure, see Section 3.2). The Co-location detector will return an indicator if it finds one or more individuals that were spatially co-located with the subject in order that the local server may inform the corresponding individual(s) for further purpose.

**Figure 3:**
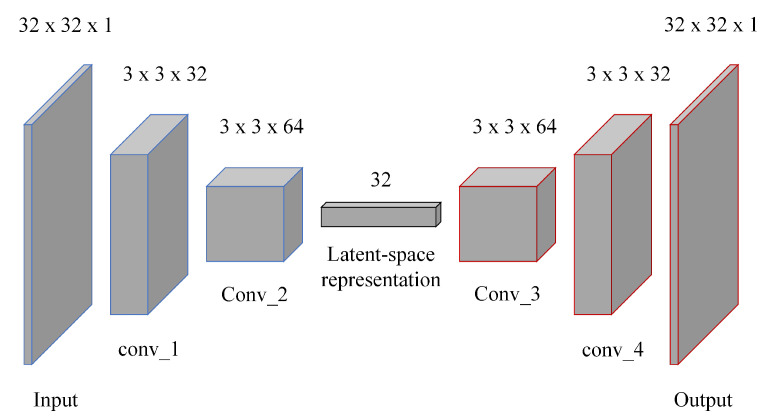
Convolutional autoencoder model inside the Co-location detector. Latent-space dimensions was set to 32×1. 32×32 input and 32×32 output correspond to the size of the training and test images.

The convolutional autoencoder model inside the co-location detector is depicted in Figure 3. Four convolutional layers were implemented. For every convolutional layer, LeakyReLU and batch normalization layers were also added. Thirty-two neurons were used for our latent space representation. Mean squared error (MSE) was still used as the objective function, which tries to minimize the difference between each pixel value of the input and each pixel value of the output (reconstructed) train images.

As mentioned by Kosasih et al. [8], the idea of using autoencoder to differentiate magnetometer readings is that after learning to reconstruct one magnetometer trajectory, if it can also reconstruct the other trajectories above a certain SSIM threshold, we can then infer that the two magnetometer data have a similar shape which thus indicates that there is a high probability that these data were collected from the same location. However, unlike the MSE analysis approach that was proposed in [8], the SSIM analysis approach could be less prone to false negative and false positive results since the SSIM index has a smaller fixed range of values compared to MSE, namely 0 to 1. This will, therefore, enable us to select a certain threshold that can be used to infer co-location of people, thus avoiding the threshold uncertainty problem faced by the MSE analysis approach in [8].

### 3.2. Spatial Co-Location Detection Procedures

**Figure 4:**
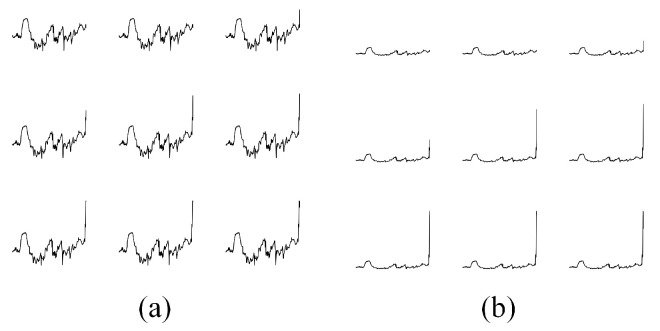
After applying tenth root operation to $\vec{F_{i}^{\prime }}$, our training and test data will look similar to (**a**). (**b**) shows training data without applying tenth root operation to $\vec{F_{i}^{\prime }}$.

**Figure 5:**
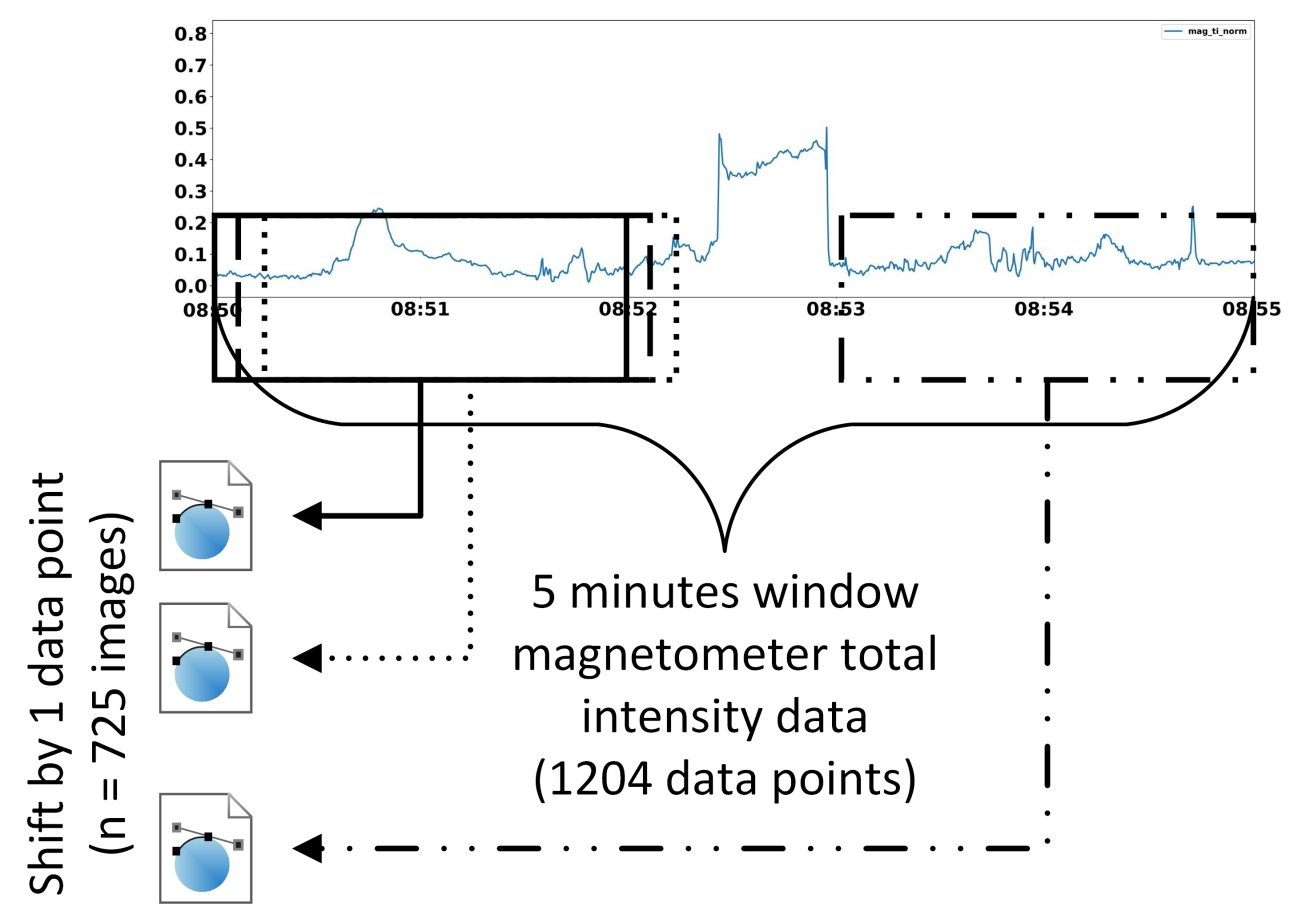
Illustration of window sliding technique used to generate training images, based on [8]. *Note*. Our window sliding technique used to generate training images is based on the window sliding technique used to generate training data matrix which was proposed by Kosasih et al. [8].

**Figure 6:**
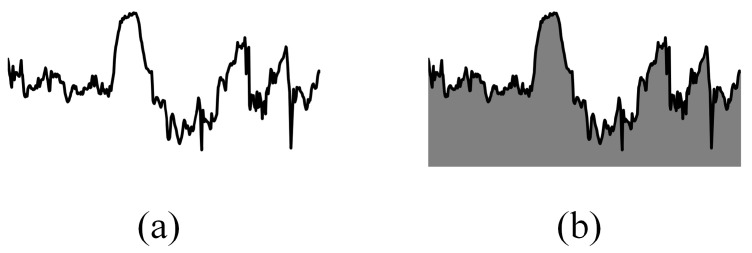
(**a**) is an example of the generated image without filling up the area under the line whereas in (**b**), we filled up the area under the line.

Let mag_x, mag_y, and mag_z be the strength of the earth’s magnetic field along 3-axes while *t* is the timestamp. Thus, the retrieved subject’s data from the main server is in the form of $S_{0}=\left(S_{0,1},\ldots ,S_{0,m}\right)$, where $S_{0,j}=\left(\vec{\text{mag\_}x_{0,j}},\vec{\text{mag\_}y_{0,j}},\vec{\text{mag\_}z_{0,j}},\vec{t_{0,j}}\right)$ represents a matrix of $j^{th}$ 5 min window during a journey of *m* windows within a specific time interval. Similarly, the retrieved other users’ data from the main server would be in the form of $\left(S_{1},\ldots ,S_{n}\right)$, where $S_{i}=\left(S_{i,1},\ldots ,S_{i,m}\right)$ represents $i^{th}$ user whose data we want to check and $S_{i,j}=\left(\vec{\text{mag\_}x_{i,j}},\vec{\text{mag\_}y_{i,j}},\vec{\text{mag\_}z_{i,j}},\vec{t_{i,j}}\right)$ represents same format with the subject’s data. The procedures inside of the Co-location detector are, therefore, as follows:

1. Data pre-processing.
  (a) The first three basic data pre-processing steps, proposed in [8], were implemented:
    - Calculating the total intensity:
      Due to the fact that mobile magnetometer readings are influenced by the device orientation, we first need to reduce the three-dimensional earth magnetic field readings into one scalar value, the total intensity (*F*). The total intensity can be found by calculating the distance between the horizontal intensity (*H*) and the vertical intensity (mag_z), while the horizontal intensity is given by the square root of the sum of the squares of the true north (mag_x) and the true east (mag_y) as can be seen in Equation (1).
      (1) $$\begin{array}{c} H_{i,k}={\left||mag_{i,k}^{z}|\right|}_{2}\\ F_{i,k}={\left||\text{mag\_}f_{i,k}|\right|}_{2} \end{array}$$
      Thus, $mag_{i,k}^{z}$ is $\left(\text{mag\_}x_{i,k},\text{mag\_}y_{i,k}\right)$ and $\text{mag\_}f_{i,k}$ would be equal to $\left(H_{i,k},\text{mag\_}z_{i,k}\right)$. *i* denotes *i*th user’s device while *k* represents *k*th data point.
    - Downsampling the total intensity:
      Each of the mobile magnetometer readings normally has various frequencies. To generate the training and test data, we would need to use the same frequency for all the data. This can be done by downsampling the total intensity of each device ($\vec{F_{i}}$) into 250 ms bins, where each new value was computed by averaging the values of the timestamps. This step would also reduce the noise, thus increasing accuracy.
    - Normalizing the total intensity:
      Next, the total intensity (*F*) was normalized such that all values are within the range of 0 and 1 to increase the model accuracy. The normalization method is formally defined in Equation (2).
      (2) $$\begin{array}{c} \vec{F_{i}^{\prime }}=\frac{\vec{F_{i}}-min\left(\vec{F_{i}}\right)}{max\left(\vec{F_{i}}\right)-min\left(\vec{F_{i}}\right)} \end{array}$$
      where $\vec{F_{i}^{\prime }}$ is the normalization result of the *i*th device.
  (b) Rescaling total intensity (F):
    After calculating the total intensity (F), downsampling *F* and normalizing it, we calculate the tenth root of each device’s normalized total intensity ($\vec{F_{i}^{\prime }}$) as can be seen in Equation (3).
    (3) $$\begin{array}{c} \vec{F_{i}^{\prime \prime }}=\sqrt[{10}]{\vec{F_{i}^{\prime }}} \end{array}$$
    where $\vec{F_{i}^{\prime \prime }}$ is the scaling result or the tenth root of $\vec{F_{i}^{\prime }}$.
    This step was implemented in order to reduce a wide range of values, thus avoiding having a flat plot as well as exposing small changes in the data.
    Figure 4 illustrates the difference between with and without applying the tenth root operation to $\vec{F_{i}^{\prime }}$. Both groups of images were generated from the same magnetometer readings. However, images in Figure 4b tends to look flat due to a wide range of values caused by outliers whereas in Figure 4a, small changes in the data are more exposed. This will help our model to learn more useful properties from the training images as well as discriminating test data. Normally, we would like to use a logarithmic scale to accomplish this task; however, since log(0) is undefined and $log(\frac{1}{x})$ where x>1 would give us a steep negative slope, we decided to use another scaler that would yield similar result to a logarithmic scale—the tenth root.
  (c) Generating training data:
    Before training our convolutional autoencoder model, we need to first generate training images instead of the training matrix which was proposed in [8], as illustrated in Figure 5. Execution steps for generating training images are as follows:
    - Generate a 32 by 32 pixels greyscale image from the first 2 min data.
    - Sift by one data point.
    - Generate the next 32 by 32 pixels greyscale image from these 2 min data (after shifting by one data point) and repeat the proses until the end of 5 min window data $\left(\vec{F_{0,j}^{\prime \prime }}\right)$. This will generate 725 training images since unlike the previous approach in [8], we do not need to repeat step i to iii for 10 times.
    There are two kinds of training images that were generated as shown in Figure 6—with and without filling up the area under the line. Our test images were also generated in the same way. By filling up the area under the line, we wish to make it more difficult for the model to reconstruct the test data as the image will have more non-zero values, thus being more decisive in distinguishing between earth’s magnetic data from the same location and earth’s magnetic data from different locations.
2. Training the Co-location detector.
  After the training data were generated using the subject’s trajectory, we feed this data into our convolutional autoencoder model inside the Co-location detector and train it. MSE was used as the objective function, which is formally defined as Equation (4).
  (4) $$MSE=\frac{1}{mn}\sum_{i=1}^{m}\sum_{j=1}^{n}{\left(\text{F\_}in_{i,j}-\text{F\_}out_{i,j}\right)}^{2}$$
  where *m* and *n* indicate the number of rows and columns in the input images, F_in is our input image and F_out is our reconstructed image.
3. Feeding other users data.
  After the training process is over, we feed other users’ data $\left(S_{1},\ldots ,S_{n}\right)$ as test data into the model and analyze the SSIM index, which is formally defined as Equation (5).
  (5) $$\begin{array}{c} SSIM\left(x,y\right)=\frac{\left(2\mu_{x}\mu_{y}+c_{1}\right)\left(2\sigma_{xy}+c_{2}\right)}{\left(\mu_{x}^{2}+\mu_{y}^{2}+c_{1}\right)\left(\sigma_{x}^{2}+\sigma_{y}^{2}+c_{2}\right)} \end{array}$$
  $\mu_{x}$ and $\mu_{y}$ are the average of *x* and *y* respectively. $\sigma_{x}^{2}$ is the variance of *x* while $\sigma_{y}^{2}$ is the variance of *y*. $\sigma_{xy}$ indicates the covariance of *x* and *y*. *x* and *y* are the location of the NXN window in each image, meaning this equation compares two windows, small sub-samples, rather than the entire images. $c_{1}={\left(K_{1}L\right)}^{2}$ and $c_{2}={\left(K_{2}L\right)}^{2}$ are variables to stabilize the division with weak denominator, with *L* and *K* being the dynamic range pixel-values and a constant variable respectively. As previously mentioned, the SSIM index is between 0 and 1, where 1 indicates perfect similarity. Therefore, we can easily set a threshold, which was 0.5 in our experiment. This means that if the score is below 0.5, the co-location detector can return an indicator to the server that this other user may have been in the same location at the same time with the subject ($S_{0}$).

## 4. Performance Evaluation

In this section, we show the experimental results of our approach and visualize the reconstructed data. We also describe the dataset used for evaluating our proposed system.

### 4.1. Dataset

We used a dataset collected by Kosasih et al. [8] in Busan, South Korea. The sensor readings were generated from three devices: Samsung Galaxy S6, Samsung Galaxy tab S5e, and Samsung Galaxy S6 edge. This dataset provided a total of three magnetometer signals; these signals are as follows:

1. Earth’s magnetic *x* positive north (mag_x) in μT
2. Earth’s magnetic *y* positive east (mag_y) in μT
3. Earth’s magnetic vertical intensity *z* (mag_z) in μT

Each reading is also accompanied by a timestamp and an activity label—still, walking, running, waiting for bus, bus, waiting for subway, subway.

**Figure 7:**
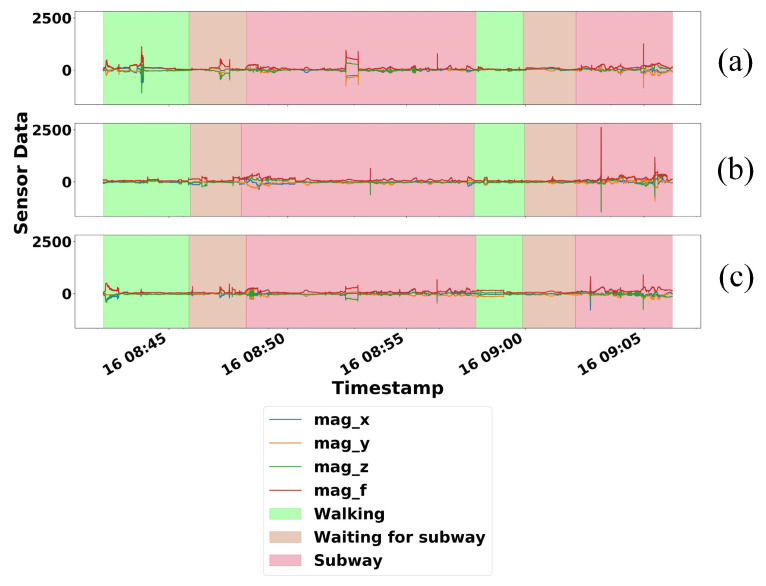
Comparison of 24 min magnetometer data from three different devices: (**a**) Samsung Galaxy S6 edge (**b**) Samsung Galaxy tab S5e (**c**) Samsung Galaxy S6 [8].

The plot of the magnetometer data provided by this dataset over a span of 24 min can be seen in Figure 7. Since they were collected together, these data should have similar shapes in general, although they have different maximum and minimum ranges of measurement.

### 4.2. Evaluation Procedures and Implementation

To evaluate our proposed system, we used magnetometer data collected from the three devices as mentioned in Section 4.1. Evaluation steps were as follows:

1. We used magnetometer data collected from Samsung Galaxy S6 edge to generate our training data. Therefore, we assumed that this device belongs to the subject.
2. We trained our convolutional autoencoder model using the generated training data.
3. We then generate the test data using magnetometer data collected from the other two devices—Samsung Galaxy tab S5e and Samsung Galaxy S6 edge.
  - Test data were generated by copying two minutes data, sifting by 240 data points (one minute), copying the next two minutes data, and repeating the process for a certain interval.
  - A total of 18 test data were generated from two devices—nine test data each. This consisted of eight test data from the same location and ten test data from different locations.
  - As shown in Figure 7, all the data were collected together while the observers were doing three different activities, namely walking, waiting for the subway, and in the subway. This indicates that the observers were always moving during the data collection activities. The only time when the observers did not move was when they waited for the subway. Since the maximum waiting time of the subway in Busan (South Korea) is five minutes, we generate different locations test data based on the minimum of five minutes difference from the training data. We assumed that by this time, the test data were collected from different places.
4. Finally, we analyzed the SSIM index to infer the co-location of these three devices. Moreover, we calculated the F1 score and generated confusion matrices to visualize the performance of our proposed system.

Our convolutional autoencoder model was implemented using Keras library with TensorFlow backend. For the gradient optimization technique, we selected ADAM [26] since it requires less memory and is well suited for large problems in terms of data and parameters. Moreover, ADAM is an adaptive learning rate algorithm; thus, it requires less tuning of the learning rate hyperparameter, making it easier to use than gradient descent. Both Sigmoid and LeakyReLU [27] were used for the activation function. Sigmoid was implemented in our output layer as we would like our output to be between 0 and 1 (normalized grayscale images). For LeakyRelu implementation, we fixed the alpha value to 0.2 since this gave us a slightly better results after running several experiments.

### 4.3. Result and Analysis

As previously mentioned, we analyzed the SSIM index to detect the co-location of two or more individuals. Two different kinds of train and test images were generated, namely dataset plot without filling up the area under the line and dataset plot with filling up the area under the line (see Section 3.2). In this section, we show the accuracy results of our proposed technique on both datasets and visualize some of the reconstructed data.

#### 4.3.1. Without Filling Up the Area under the Line

**Figure 8:**
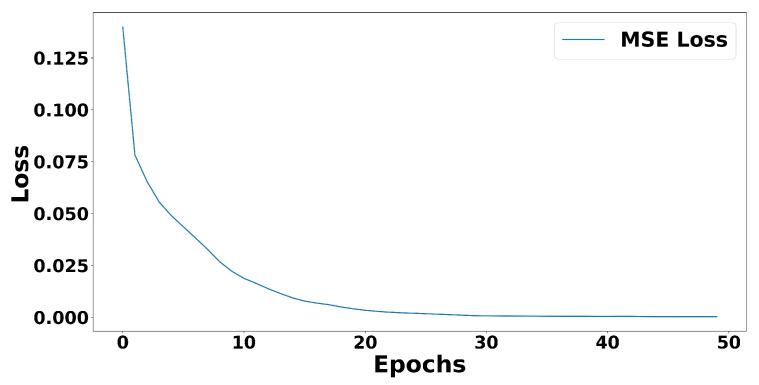
MSE loss during convolutional autoencoder model training (without filling up the area under the line). It started to converge after 15 epochs.

Our model only took 15 epochs, approximately 9.53 s, to converge, as can be seen in Figure 8 when trained on NVIDIA GeForce RTX 2080 Ti (11 GB). If we multiply it by 288 derived from $\frac{60}{5}\times 24$ for 24 h data, it will only take approximately 45.744 min to train the whole one-day data. This implies that compare to training time in [8], it would take twice as long to train our convolutional autoencoder model on this dataset (without filling up the area under the line).

**Figure 9:**
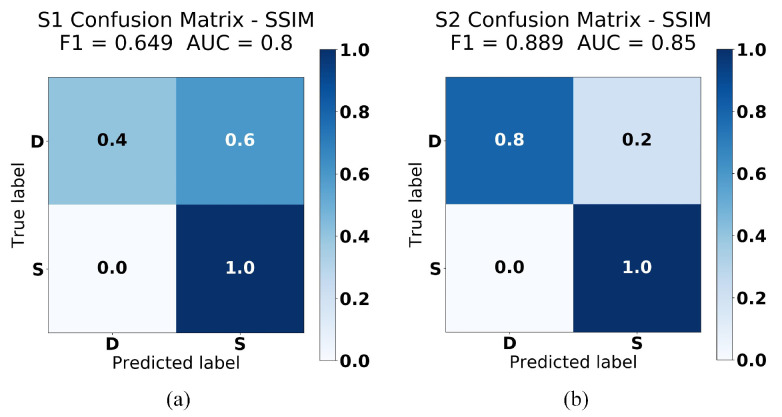
Confusion Matrices of SSIM analysis experiment (without filling up the area under the line) on (**a**) test data 1: Samsung Galaxy tab S5e (**b**) test data 2: Samsung Galaxy S6.

Figure 9 visualize the performance of our SSIM analysis approach trained on the dataset plot without filling up the area under the line. F1 score on the first (Figure 9a) and second (Figure 9b) test data, collected from our two test devices, were 0.65 and 0.89 respectively—slightly lower than F1 score of the MSE analysis approach in [8] on the first test data. In addition, we also calculated AUC (area under the curve) to assess the performance of our system. AUC scores on the first (Figure 9a) and second (Figure 9b) test data, were 0.8 and 0.85 respectively. This indicates that there is more than 80% chance that this approach will be able to distinguish between co-located and non-collocated users. Although it seems that our SSIM analysis approach performed worse than the MSE analysis approach, in terms of F1 score, this approach is actually less prone to false positive or false negative as SSIM indexes only range from 0 to 1, meaning we have a relatively smaller fixed range of values to be selected as our threshold. As can be seen in Table 1, we selected 0.5 as our threshold since it gave us the best classification performance.

Table 1 shows the individual SSIM index from our experiment on this dataset. As can be seen in this table, our system misclassified three test data from the first device, whereas only one test data were misclassified from the second device.

**Figure 10:**
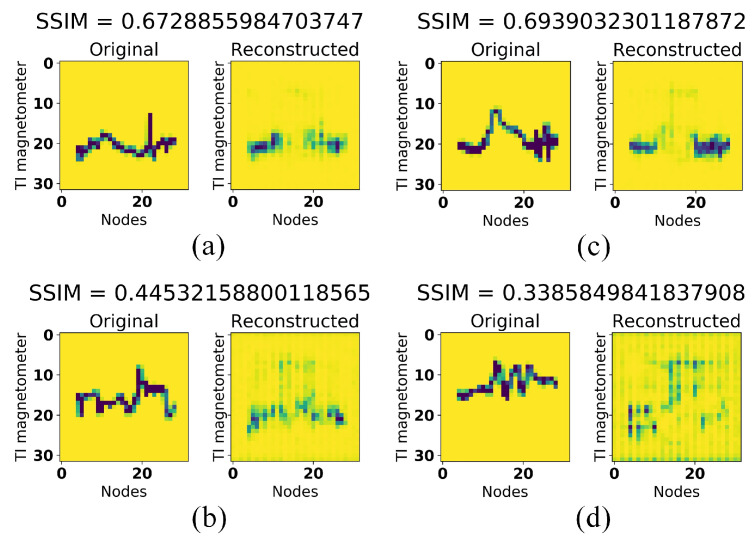
Test data reconstruction results of SSIM analysis experiment (without filling up the area under the line) when (**a**,**c**) the test devices were in the same location with the training device (**b**,**d**) the test devices were in the different location with the training device. (**a**,**b**) were collected from Samsung Galaxy tab S5e, whereas (**c**,**d**) were collected from Samsung Galaxy S6.

We also display some reconstruction results of this experiment as can be seen in Figure 10. Figure 10a,b are reconstruction results from the first test data whereas Figure 10c,d are reconstruction results from the second test data. Test data in Figure 10a,c were collected from the same location with the training data while Figure 10b,d were collected from different locations. These results indicate that fixing SSIM index to 0.5 would give us best classification performance.

#### 4.3.2. Filling Up the Area under the Line

**Figure 11:**
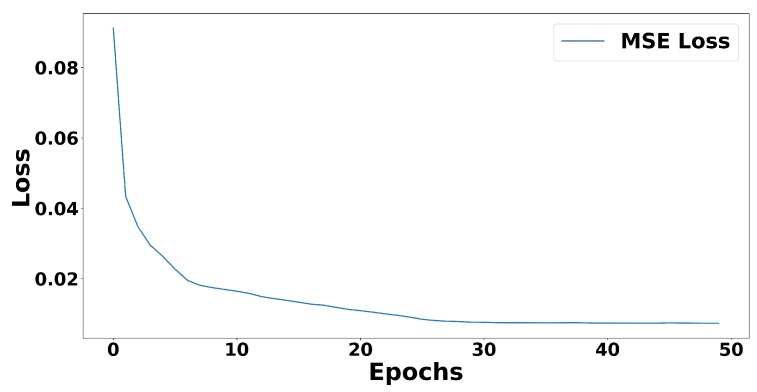
MSE loss during convolutional autoencoder model training (filling up the area under the line). It started to converge after ten epochs.

When we trained our convolutional autoencoder model on this dataset, it took ten epochs, approximately 7.28 s, for our model to converge, as can be seen in Figure 11. This indicates that it would take around 34.944 min to train 24 h data. This training duration is similar to the training duration in [8].

**Figure 12:**
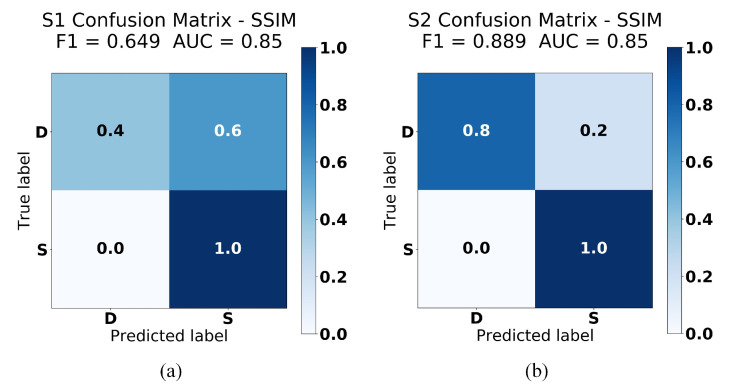
Confusion Matrices of SSIM analysis experiment (filling up the area under the line) on (**a**) test data 1: Samsung Galaxy tab S5e (**b**) test data 2: Samsung Galaxy S6.

Figure 12 visualize the performance of our SSIM analysis approach trained on the dataset plot with filling up the area under the line. As can be seen from this figure, there was not any change in the F1 scores compared to the above approach (Section 4.3.1—without filling up the area under the line). The AUC scores, however, were 0.85 on both test data, which suggests that there is more than 85% chance that our second approach will be able to distinguish between co-located and non-collocated users. This indicates that adding more non-zero values to our train and test images data will not significantly improve the performance of our model, as can also be seen in Table 2. This table shows that the SSIM indexes of this experiment were similar to our experiment in Section 4.3.1, except for the test data that were collected from the same location. We could see that with this approach, the SSIM index would yield higher values which could lead to a fewer number of false negative. Moreover, experiment results on Table 1 and Table 2 show that our algorithm performed less effectively on the Samsung Galaxy tab S5e magnetic readings. This is particularly true for non-co-located users as it managed to reconstruct the sensor readings from different spatial locations. Our hypothesis is that it was caused by the use of more robust sensors in smartphones than in tablets, as was also shown in the smartphones and tablets’ sensors precision study by Novakova et al. [28]. However, we would need to conduct more experiments with more datasets in order to prove these hypotheses.

**Figure 13:**
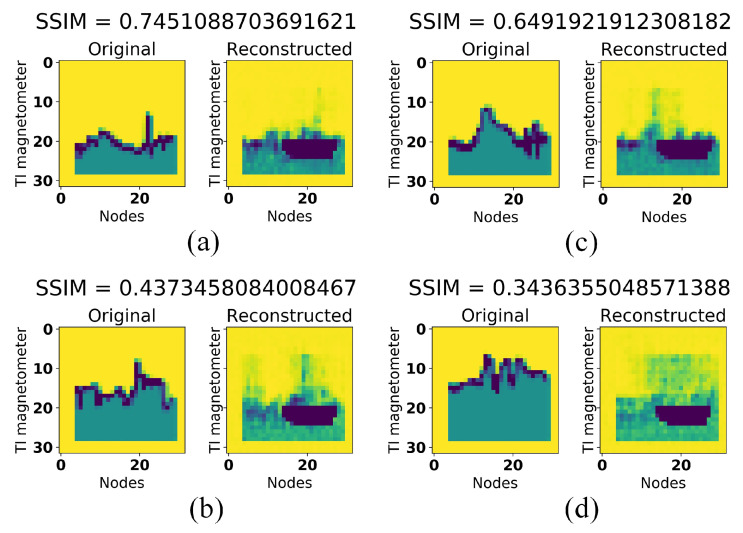
Test data reconstruction results of SSIM analysis experiment (filling up the area under the line) when (**a**,**c**) the test devices were in the same location with the training device (**b**,**d**) the test devices were in the different location with the training device. (**a**,**b**) were collected from Samsung Galaxy tab S5e, whereas (**c**,**d**) were collected from Samsung Galaxy S6.

Figure 13 depicts some reconstruction results of this experiment. Figure 13a,b are reconstruction results from the first test data whereas Figure 13c,d are reconstruction results from the second test data. Test data in Figure 13a,c were collected from the same location with the training data while Figure 13b,d were collected from different locations. In these figures, we could better observe how adding more non-zero values to our train and test images may lead to slightly higher or lower SSIM index (wider SSIM indexes gap between same location test data and different location test data).

## 5. Conclusions

The spatial co-location detection task is a crucial concept that has many applications in various fields. In this paper, we proposed the implementation of an unsupervised learning algorithm to analyze mobile magnetometer readings with the express purpose of inferring the co-location of two or more individuals. The novelty of our idea is that using the SSIM index to analyze the reconstructed data in the form of images, we will be able to set a certain threshold from which the co-location of people can be deduced. This approach also enables us to avoid multiple comparison processes since we are only required to train our model once and employ it to analyze as many other mobile magnetometer readings as we want. The concept of fog computing was also proposed in this work to alleviate hardware limitation problems and improve computational speed while still preserving low latency communications.

We evaluated our proposed system using a dataset that was collected in Busan, South Korea. The evaluation results indicated an accuracy of 65–90% in recognizing the co-location of two or more individuals within a specific time interval in our test environment. Our future work would be to solve 5 min windows data limitation due to temporal problems. This is because the efficiency of this system could be improved by increasing the amount of data that can be trained at one time, such as 24 h data. Moreover, although the concept of using magnetometer readings to infer co-location of people may alleviate excessive power consumption problems on the mobile devices due to the use of GPS, it is still prone to one essential issue—that is, the system would be easily fooled by distracting the earth’s magnetic field readings using other magnetic devices. Therefore, further research on solving this drawback would be essential for our system.

## Figures and Tables

**Table 1:** Experiment results on SSIM analysis method (without filling up the area under the line) with “S” being the same location while “D” being different location. Misclassified results are highlighted in grey.

No	Test Data 1: Samsung Galaxy Tab S5e (Threshold: 0.5)			Test Data 2: Samsung Galaxy S6 (Threshold: 0.5)		
	SSIM	SSIM Prediction	Ground Truth	SSIM	SSIM Prediction	Ground Truth
0	0.585592	S	S	0.693903	S	S
1	0.748521	S	S	0.533133	S	S
2	0.672886	S	S	0.501918	S	S
3	0.659999	S	S	0.522440	S	S
4	**0.609034**	**S**	**D**	**0.618838**	**S**	**D**
5	**0.715449**	**S**	**D**	0.427649	D	D
6	**0.504019**	**S**	**D**	0.393774	D	D
7	0.468111	D	D	0.309711	D	D
8	0.445322	D	D	0.338585	D	D

**Table 2:** Experiment results on SSIM analysis method (filling up the area under the line) with “S” being the same location while “D” being different location. Misclassified results are highlighted in grey.

No	Test Data 1: Samsung Galaxy Tab S5e (Threshold: 0.5)			Test Data 2: Samsung Galaxy S6 (Threshold: 0.5)		
	SSIM	SSIM Prediction	Ground Truth	SSIM	SSIM Prediction	Ground Truth
0	0.697742	S	S	0.649192	S	S
1	0.812677	S	S	0.513390	S	S
2	0.745109	S	S	0.514484	S	S
3	0.697573	S	S	0.511380	S	S
4	**0.667691**	**S**	**D**	**0.566473**	**S**	**D**
5	**0.789856**	**S**	**D**	0.433923	D	D
6	**0.569859**	**S**	**D**	0.304871	D	D
7	0.471322	D	D	0.297473	D	D
8	0.437346	D	D	0.343636	D	D

## Data Availability

Publicly available datasets were analyzed in this study. This data can be found here: https://www.kaggle.com/davidishakkosasih/magnetometer-datasets (accessed on 13 July 2021).

## Abbreviations

The following abbreviations are used in this manuscript:

SSIM	Structural Similarity
MSE	Mean Squared Error
